# The role of PfEMP1 as targets of naturally acquired immunity to childhood malaria: prospects for a vaccine

**DOI:** 10.1017/S0031182015001274

**Published:** 2016-01-08

**Authors:** PETER C. BULL, ABDIRAHMAN I. ABDI

**Affiliations:** 1 Department of Pathology, University of Cambridge, Tennis Court Rd, Cambridge CB2 1QP, UK; 2 Nuffield Department of Medicine Research Building, Centre for Tropical Medicine and Global Health, University of Oxford, Old Road Campus, Roosevelt Drive, Headington, Oxford OX3 7FZ, UK; 3 Department of Biochemistry and Chemistry, Pwani University, P.O. Box 195, 80108 Kilifi, Kenya

**Keywords:** malaria, immunity, vaccine, variant surface antigens, PfEMP1

## Abstract

The *Plasmodium falciparum* erythrocyte membrane protein 1 antigens that are inserted onto the surface of *P. falciparum* infected erythrocytes play a key role both in the pathology of severe malaria and as targets of naturally acquired immunity. They might be considered unlikely vaccine targets because they are extremely diverse. However, several lines of evidence suggest that underneath this molecular diversity there are a restricted set of epitopes which may act as effective targets for a vaccine against severe malaria. Here we review some of the recent developments in this area of research, focusing on work that has assessed the potential of these molecules as possible vaccine targets.

## INTRODUCTION

When *Plasmodium falciparum* infect human erythrocytes, they insert into the erythrocyte surface parasite antigens that profoundly alter the antigenic properties of the cells (Langreth and Reese, 1979). These antigens play a central role in the pathology of severe malaria by mediating the cytoadhesion to various molecules on host cells (Rowe *et al.* 2009) and sequestration of parasites in tissues including the brain, long known as a hallmark of fatal falciparum malaria in humans (Marchiafava and Bignami, 1894). One of their key features is that they undergo antigenic variation (Roberts *et al.* 1992) and for this reason they are collectively called variant surface antigens (VSA).

The feasibility of developing a malaria vaccine is supported by the observation that children growing up in malaria endemic areas develop naturally acquired immunity to malaria after several years of exposure (Wilson *et al.* 1950). This immunity protects children from severe life threatening malaria and promotes the establishment of chronic, asymptomatic infections, to which even individuals growing up in malaria endemic areas remain susceptible for life (Marsh, 1992). In this review, we will focus on the role of the *P. falciparum* membrane protein 1 (PfEMP1) family of VSA as targets of naturally acquired immunity and review their potential as vaccine targets. There have been extremely encouraging developments in this area of research over the last 3 years. However, as our knowledge grows, so does our appreciation of the complexity of the parasite's strategy of evading our immune systems. There are no shortage of recent reviews that together give a comprehensive summary of variants surface antigens both in immunity and cytoadhesion (Rowe *et al.* 2009; Chan *et al.* 2014; Smith, 2014). Here, we will give an overview of what we think are the current key questions and gaps in our knowledge.

### VSA are important targets of naturally acquired immunity

An early demonstration of the importance of VSA as immune targets was a serological study showing that, among seven different anti-blood stage assays including one measuring opsonization of infected erythrocytes, the titre in children's serum of antibodies that agglutinated infected erythrocytes from a single donor, was the only assay associated with reduced future experience of clinical malaria (Marsh *et al.* 1989). This apparent protection by anti-VSA antibodies against future disease is supported by more recent longitudinal studies by Dodoo *et al.* (2001), where antibodies to infected erythrocytes of 2/5 parasite isolates showed a clear association with protection after age correction. Measures of antibodies to the infected erythrocyte surface present at the time of infection also show a negative association with the severity of disease, supporting a role in immunity (Tebo *et al.* 2002; Yone *et al.* 2005). Consistent with this, chronic asymptomatic infections are associated with particularly high levels of antibodies to VSA and individuals who carry asymptomatic infections but do not make such responses tend to be more susceptible to future clinical malaria (Bull *et al.* 2002; Kinyanjui *et al.* 2004; Mackintosh *et al.* 2008). The third approach used in early epidemiological studies was to test for carriage of antibodies that reverse cytoadhesion. The rosetting phenotype, defined as the binding of infected erythrocytes to uninfected erythrocytes, provides a good example. Carlson *et al.* (1990) found an association between the presence in children's serum of antibodies that reverse rosetting of a laboratory-adapted parasite line and (1) reduced rosetting in the children's own parasites and (2) absence of cerebral malaria. Antigen variants expressed on the surface of parasites from children with malaria was later shown to correspond with gaps in the repertoire of antibodies carried by the infected individual before they became ill (Bull *et al.* 1998; Giha *et al.* 2000). Together, these data support a model of naturally acquired immunity to malaria through the gradual acquisition of many variant specific antibodies to VSA.

Such a model is consistent with the known dynamics of *P. falciparum* infections through information gathered during the time when malaria was used in the therapy of neurosyphilis (Whitrow, 1990; Karamanou *et al.* 2013). The importance of VSA as targets of naturally acquired immunity to malaria is highlighted by the observation of clear recrudescence in parasitaemia followed by rapid decline. When these recrudescence occured, they did so with relatively consistent initial growth rates suggesting that new variants were emerging and were subsequently controlled on an otherwise only gradually changing immunological background (Molineaux *et al.* 2001).

*Plasmodium falciparum* encodes several multi-gene families including *var, rif* and *stevor* for which there is evidence of expression on the surface of parasite-infected erythrocytes (Baruch *et al.* 1995; Smith *et al.* 1995; Su *et al.* 1995; Cheng *et al.* 1998; Fernandez *et al.* 1999; Kyes *et al.* 1999; Niang *et al.* 2014; Goel *et al.* 2015). *Rif* and *stevor* together with the majority of the 60 genes that make up the *var* gene family are located in the highly diverse sub-telomeric regions of chromosomes (Gardner *et al.* 2002). Of these families the *var* genes and the PfEMP1 antigens they encode have been the subject of the most research to date because of the role that PfEMP1 play both in cytoadhesion to many specific host receptors and the evidence for their role as targets of naturally acquired antibodies that undergo clonal antigenic variation.

An early study demonstrated how changes in PfEMP1 expression by a laboratory parasite line correlated with switches in both the antigenic and cytoadhesive properties of the infected erythrocyte surface (Biggs *et al.* 1991). After the *var* genes had been identified (Baruch *et al.* 1995; Smith *et al.* 1995; Su *et al.* 1995), switches in *var* gene expression were correlated with both antigenic variation and altered ability of the parasites to bind to the host receptor molecule ICAM1 (Smith *et al.* 1995). Many known host-cell-binding phenotypes identified in *P. falciparum*-infected erythrocytes have since been mapped onto regions of PfEMP1, including ICAM1 (Smith *et al.* 2000; Bengtsson *et al.* 2013), CD36 (Baruch *et al.* 1997; Smith *et al.* 1998; Robinson *et al.* 2003), complement receptor 1(Rowe *et al.* 1997), heparin, blood group A antigen (Vigan-Womas *et al.* 2012) , Chondroitin sulphate A (Salanti *et al.* 2003), PECAM1 (Berger *et al.* 2013), IgM (Ghumra *et al.* 2008) and endothelial protein C receptor (EPCR) (Turner *et al.* 2013). Recently, knockdowns of *var* gene expression in two laboratory lines resulted in almost complete loss of antibody recognition and cytoadhesive properties (Chan *et al.* 2012), suggesting that PfEMP1 are the dominant antigens expressed on the surface of these laboratory-adapted parasite lines that are recognized by naturally acquired antibodies against infected erythrocytes.

### Why is the feasibility of a PfEMP1 vaccine worth exploring?

PfEMP1 clearly plays an important role in the host–parasite interaction. However, PfEMP1 is, on the face of it, perhaps the last group of proteins that you might want to study as a malaria vaccine candidate. Early serological studies show that after an episode of disease, children develop antibodies that are highly specific to the parasites that caused that disease episode, suggesting that, at least the immune-dominant epitopes expressed on the infected erythrocyte surface are extremely diverse (Marsh and Howard, 1986; Forsyth *et al.* 1989; Iqbal *et al.* 1993; Reeder *et al.* 1994; Bull *et al.* 1999). Though they are encoded by a family of only 60 *var* genes in every genome, each genome is potentially a drop in the ocean of *var* diversity within the global parasite population and the potential for immune evasion through influx of new PfEMP1 variants through gene conversion would seem from these data to be immense. Barry *et al*. (2007), sampled short tags of sequence from PfEMP1 from a worldwide collection of parasites and failed to find a limit to this sequence diversity. As new sequences were sampled randomly from the ‘pot’ of collected sequences, a point was never reached at which the rate of sampling of new sequences diminished (Barry *et al.* 2007). Recent evidence further suggests that *var* undergo mitotic recombination, potentially unpacking unlimited diversity from a single genome (Claessens *et al.* 2014).

Despite this immense molecular diversity children as they grow up in malaria endemic areas do learn to recognize antigens expressed on the surface of infected erythrocytes (Barragan *et al.* 1998; Bull *et al.* 1998) and cross-reactivity exists between epitopes expressed on the surface of infected erythrocytes sampled from different geographical areas and even different continents (Aguiar *et al.* 1992; Nielsen *et al.* 2004). So why does antigenic variation in *P. falciparum* not always lead to infections that overwhelm the host? The life cycle of *P. falciparum* relies on the establishment of chronic blood stage infections and host survival through the dry season when pools of water necessary for mosquito reproduction are scarce and opportunities to transmit to mosquitos is low. The question of how the correct balance is maintained between too little and too much antigenic diversity; host survival and parasite escape from antibodies, is still one of the major questions in malaria parasite biology (Saul, 1999). The generally accepted broad explanation is that antigenic diversity in PfEMP1 is constrained by its function in cytoadhering to host cells and bringing about sequestration of infected erythrocytes in tissue capillary beds and that inefficient cytoadhesion leads to passage of the infected erythrocytes through the spleen where they are removed from circulation (Barnwell *et al.* 1983).

**Fig. 1.**
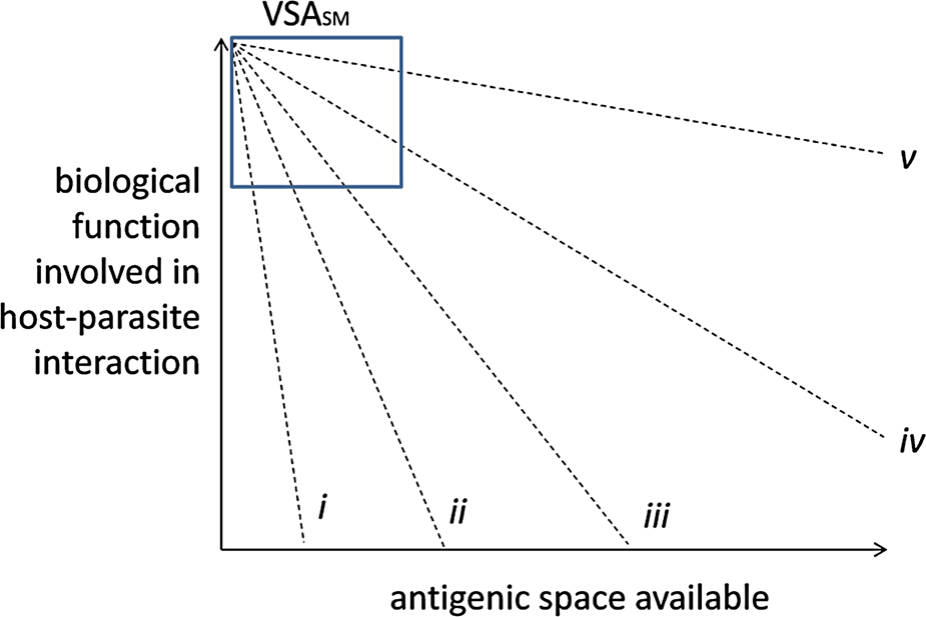
Hypothetical trade-offs between function and antigenic novelty. This figure shows an immunological version of the principle of functional constraint focusing on efficiency of biological functions associated with the host–parasite interaction (*y*-axis) and the hypothetical antigenic space within which those functions exist (*x*-axis). Optimal function can only be performed by a narrow range of molecular structures which translates, in the simplest case, to a small antigenic space. Key to virulence is not simply the function itself, but the ability of molecules or systems of molecules to uncouple immunogenicity from function to expand the antigenic space available. Hypothetical lines are drawn to suggest trade-offs between function and antigenic space in for: (i) Measles haemagglutinin (Frank and Bush, 2007), (ii) var2CSA, (iii) group A and DC8 PfEMP1 (Buckee and Recker, 2012), (iv) group B and C PfEMP1, (vi) HIV gp120 (de Boer and Boerlijst, 1994). Following from this hypothetical trade-off, parasites with high levels of recognition by antibodies commonly carried by children have been proposed to express PfEMP1 variants with strong cytoadhesive function and exist within a small restricted immunological space. Such hypothetical variants have been called: agglutination frequency high (AF^H^ (Bull *et al.* 2000) VSA with a high frequency of recognition [VSA_FoRH_ (Bull *et al.* 2005*b*)], and VSA associated with severe malaria [VSA_SM_ (Nielsen *et al.* 2002; Jensen *et al.* 2004)]. Their position within the graph is indicated with a box

The idea that the functional role of PfEMP1 constrains its antigenic diversity forms an important part of the rationale for considering PfEMP1 as a viable vaccine target against severe malaria (see Fig. 1). This rationale stems from the way in which naturally acquired immunity develops. Children who grow up in malaria endemic areas probably never develop sterile immunity to *P. falciparum*. Immunity to severe malaria develops more rapidly than immunity to mild malaria as the host–parasite relationship changes from one associated with severe malaria and transmission to mosquitoes towards one that sustains chronic asymptomatic infections (Langhorne *et al.* 2008; Goncalves *et al.* 2014; Griffin *et al.* 2015). If constraints on PfEMP1 structure are imposed by effective cytoadhesion and avoidance of the host spleen, this could result in trade-off between antigenic novelty on the one hand and highly effective cytoadhesion on the other (Bull *et al.* 1999; Nielsen *et al.* 2002; Jensen *et al.* 2004; Frank and Bush, 2007; van Noort *et al.* 2010; Buckee and Recker, 2012; Severins *et al.* 2012). If only strongly cytoadhesive PfEMP1 can mediate the level of sequestration that can bring about severe malaria and these variants are more structurally constrained and antigenically conserved, then it is possible to imagine this trade-off setting up a spectrum of variants differentially adapted to efficient cytoadhesion on the one hand and antibody evasion on the other. The observed shift in host–parasite interaction observed in children growing up exposed to malaria parasites could be brought about through rapid acquisition of immunity to the few types that support high levels of cytoadhesion and differential survival of parasites expressing variants that can only support uncomplicated and asymptomatic infections.

Evidence for this model comes first from the association between various cytoadhesion phenotypes and the severity of malaria (Carlson *et al.* 1990; Rowe *et al.* 1995; Newbold *et al.* 1997; Heddini *et al.* 2001; Ochola *et al.* 2011; Turner *et al.* 2013). The second line of evidence comes from studies that have examined variation in the serological commonness of antigens expressed on the surface of infected erythrocytes measured by panels of plasma or serum. Parasites from children with severe malaria and those who were very young tend to be more commonly recognized (Bull *et al.* 1999, 2000; Nielsen *et al.* 2002). The fact that severe malaria is a rare manifestation of infection seems at odds with the idea that it is associated with commonly occurring variants and raises the question of why some children get severe malaria and others don't. We might hypothesize that the expression of subsets of commonly circulating PfEMP1 is necessary, if not sufficient, for the development of severe malaria. However, this would be at odds with the observation that parasites from 13/42 children with severe malaria in (Bull *et al.* 2000) were not agglutinated by any of a panel of 15 children's plasma (Bull *et al.* 2005*b*). The serological profiles of commonly recognized parasites were also diverse showing that there may be many commonly recognized types circulating in the parasite population (Bull *et al.* 1999, 2000, 2005*b*; Nielsen *et al.* 2002).

### PfEMP1 structure reflects the tension between cytoadhesive function and antigenic novelty

**Fig. 2.**
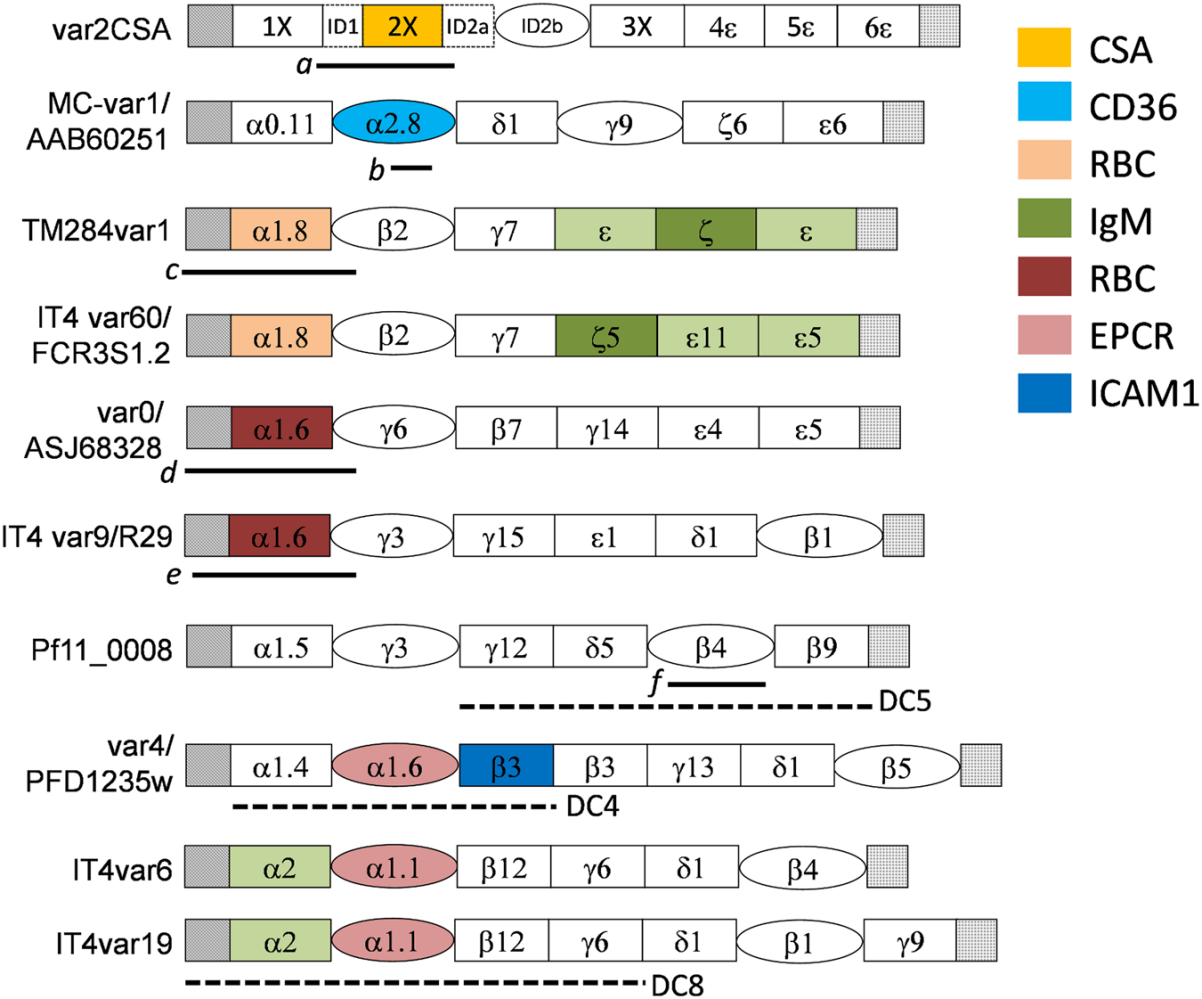
The structures of some notable PfEMP1 variants. Duffy-binding domains (DBL) are shown as rectangles and cysteine-rich interdomain regions (CIDR) and the CIDR-like domain of var2CSA are shown as ovals, N-terminal segment and acidic terminal segment (ATS) sequences are shown as hatched and stippled squares, respectively. ID2a and ID2b are interdomain regions within var2CSA that from part of the DBL2X CSA-binding domain (Clausen *et al.* 2012). Cytoadhesion phenotypes associated with specific domains are shown as colours. Recombinant proteins derived from these PfEMP1 are shown as black horizontal lines under the region of the molecules from which they were derived a, b, c, d, e, f. Domain cassettes are shown as horizontal dashed lines: a, the Id1-DBL2X region can stimulate antibodies in mice that inhibit CSA binding (Bordbar *et al.* 2012); b, The r179 region of CD36-binding CIDR from the Malayan Camp line used by Baruch *et al.* (1997) to induce homologous protection in *Aotus* monkeys; c, the region of the IgM binding, rosette mediating TM284var1 used by Ghumra *et al.* (2012) to induce cross-reactive, opsonizing antibodies in rabbits; IT4var60 was found to be the rosette mediating *var* in the well-studied cell line FCR3S1·2 (Albrecht *et al.* 2011); d,e, constructs from non-IgM-binding, rosette-mediating PfEMP1 found to induce non-cross-reactive, rosette-inhibiting antibodies (Vigan-Womas *et al.* 2011); (Ghumra *et al.* 2012); The R29 line was recently found to simultaneously form rosettes and bind to human brain endothelial cells (Adams *et al.* 2014); f, naturally acquired antibodies to the DBL*β*4 domain of pf11_0008 were associated with protection from future malaria in a longitudinal study (Magistrado *et al.* 2007). A PECAM1-binding domain cassette (DC5) was subsequently identified within this gene (Berger *et al.* 2013). Also shown are DCs, DC4 found to bind ICAM1 (Bengtsson *et al.* 2013), identified within *var4* previously found to be dominantly expressed in parasites selected for binding to antibodies from semi-immune children (Jensen *et al.* 2004). Note the additional presence of a CIDR*α*1.6 within *var4*, shown to bind to EPCR by Lau *et al.* (2015) but not by Turner *et al.* (2013). IT4var6 and IT4var19 were two genes selected for binding to cells lines derived from human brain endothelial cells (Avril *et al.* 2012; Claessens *et al.* 2012), within which the DC8 and the EPCR-binding CIDRa1·1 domain was identified (Turner *et al.* 2013).

The structure of PfEMP1 nicely reflects its dual role as an antigenically variant cytoadhesive molecule. Despite considerable molecular diversity and length variation between the PfEMP1 variants, there is underlying structural conservation at various levels. This can be summarized as follows: (1) the molecules are constructed from only two broad classes of domain [see (Higgins and Carrington, 2014) for a recent review]. The most common type of domain, ‘duffy binding-like’ (DBL) exists in several forms in *Plasmodium* species, in the duffy blood group binding protein in *Plasmodium vivax*, and in *P. falciparum*, the EBA140 and EBA175 erythrocyte-binding antigens and in the MSPDBL2 merozoite surface protein. The other domain type found in PfEMP1 is called the ‘cysteine-rich interdomain region’ (CIDR). (2) Both CIDR and DBL contain blocks of sequence (homology blocks) that are relatively conserved. These homology blocks can be used to divide each domain type into various classes and subclasses (Rask *et al.* 2010). (3) The key feature of these domain subclasses is that they represent deep divisions within the sequences. These broad classes of domain are commonly encountered between different parasite isolates and antigenic differences found within each parasite genome appear to be broadly conserved (Joergensen *et al.* 2006, 2007). This is reminiscent of the deep divide seen within MHC genes (Klein and O'Huigin, 1994) and is supported by the existence of a similar range of common sequence features in *var* sequences from *Plasmodium reichenowi*, a parasite species similar to *P. falciparum* that infects chimpanzees (Bull *et al.* 2008; Zilversmit *et al.* 2013; Otto *et al.* 2014). In the case of PfEMP1 domains, specific cytoadhesive phenotypes are associated with specific domain subtypes (Fig. 2 and Table 1). (4) Finally, the predicted three-dimensional structures of these domains tends to be conserved and PfEMP1 molecules associated with childhood malaria are thought to exist as loose modular string of domains each with one of two basic structures (Klein *et al.* 2008; Higgins and Carrington, 2014).

**Table 1.** Immunological studies on PfEMP1 domains

DC	Key domains	SD	HB	Motif	var	Adhesion	SM	C/R Ab	P.reich.	Antibodies	Surface	Protection	References
DC11	NTS-DBL*α*1·8/DBL*ζ*					Rosetting/IgM		Yes		Rabbits	Yes	Opsonization	[1]
DC16	NTS-DBL*α*1·5/DBL*ζ*				HB3VAR06	Rosetting/IgM		Yes		Rabbits	Yes	Opsonization	[1, 2]
DC5					Pf11_0008	PECAM		No	Yes	Rat	Yes	Longitudinal	[3]
DC4	CIDR*α*1·6/DBL*β*3-D4				var4	ICAM1/EPCR	Yes	Yes	Yes	Rat/human	Yes	IE adhesion	[4–7]
DC13	CIDR*α*1·4					EPCR	Yes	Yes		Human	Yes		[8–10]
DC8	CIDR*α*1·1				IT4var19	EPCR	Yes	Yes		Human	Yes		[8–11]
	CIDR*α*1·1					EPCR		Yes		Human	Yes		[6, 11]
	CIDR*α*1·4					EPCR		Yes		Human	Yes		[6, 11]
	CIDR*α*1·5					EPCR		Yes		Human	Yes		[6]
	CIDR*α*1·8					EPCR		Yes		Human	Yes		[6]
	CIDR*α*2.8				MC-var1	CD36		Yes		Aotus	No	Vaccination	[12]
	CIDR*β*4				Pf11_0008			Yes		Human	Yes	Longitudinal	[13]
	NTS-DBL*α*1·6	1,2			varO	Rosetting		No		Human/mice	Yes		[14, 15]
	NTS-DBL*α*1·8	3			IT4var60	Rosetting		Yes		Mouse	Yes	Anti-roset	[16]
	NTS-DBL*α*1·8	1,2			IT4var60			Yes		Human		Anti-roset	[17]
	NTS-DBL*α*1·8	2–3	~2	DYVPQ(F/Y)LR				Yes		Human		Anti-roset	[17]
	NTS-DBL*α*1·8	2	5	RDSM(ALNRKE)			RD	Yes		Human/rat/rabbit	Yes		[18, 19]
		1	4	GACxPxRRxxLC		C32 cells		Yes		Aotus	Yes	Vaccination	[20]

DC, domain cassette; SD, sub-domain; HB, homology block; *var*, commonly used model *var*; SM, association between expression and disease severity; C/R Ab, Whether cross-reactive antibodies have been detected; *P. reich*., identification of structure in *Plasmodium reichenowi*; antibodies, source of antibodies used; protection, the method used to demonstrate that antibodies are protective; longitudinal, longitudinal cohort studies of human populations; adhesion, reversal of infected erythrocyte adhesion; vaccination, protection demonstrated by vaccinating animals; anti-roset, rosette disruption by antibodies. RD, malaria with respiratory distress.

References: 1. Ghumra *et al.* (2012); 2. Stevenson *et al.* (2014); 3. Berger *et al.* (2013); 4. Jensen *et al.* (2004); 5. Bengtsson *et al.* (2013); 6. Lau *et al.* (2015); 7. Janes *et al.* (2011); 8. Avril *et al.* (2012); 9. Claessens *et al.* (2012); 10. Lavstsen *et al.* (2012); 11. Turner *et al.* (2013); 12. Gamain *et al.* (2001); 13. Magistrado *et al.* (2007); 14. Vigan-Womas *et al.* (2011); 15. Vigan-Womas *et al.* (2012); 16. Angeletti *et al.* (2012); 17. Albrecht *et al.* (2014); 18. Normark *et al.* (2007); 19. Blomqvist *et al.* (2013); 20. Patarroyo *et al.* (2014).

### Functional specialization is supported by serological and expression studies

A single clear example shows how effectively evolution has shaped the PfEMP1 repertoire to adapt both immunologically and functionally to its host. Malaria in pregnancy is associated with a single PfEMP1 variant encoded by var2CSA (Salanti *et al.* 2003, see Box 1). This PfEMP1 binds specifically to chondroitin sulphate A but not to CD36 and is adapted to bind to syncitiotrophoblasts in the placenta. Parasites isolated from placenta are poorly recognized by antibodies from children, male adults and women during their first pregnancy but immunity develops during subsequent pregnancies, explaining the observed parity dependency in susceptibility to placental malaria (Beeson *et al.* 1999).

Box 1. Lessons from VAR2CSA.

VAR2CSA presents us with what on the surface seemed like the best case scenario for developing a PfEMP1-based vaccine to targeting severe malaria. This has been more challenging perhaps than the expected but this should not be used as evidence that a vaccine based on other, more variable PfEMP1 involved in childhood malaria will be substantially more difficult to achieve.

The reason is that, unlike PfEMP1 associated with childhood malaria, VAR2CSA forms a compact structure, possibly due to the nature of the molecules to which it binds and the immunogenicity of the entire molecule is very different from the individual domains (Higgins and Carrington, 2014). This difference with other PfEMP1 is consistent with the fact that var2CSA is not genetically connected to the network of gene conversion events that occur between other *var* genes. If all *var* genes had compact structures these would be disrupted each time the genes recombined with other *var* genes. A modular structure is likely to be more robust to their mode of variation through recombination.

One specific challenge in the case of VAR2CSA is that naturally acquired immune responses tends to target regions DBL3X and DBL5e that are masked by their ability to bind non-specifically to IgM (Barfod *et al.* 2011). Though some PfEMP1 associated with childhood malaria bind non-specifically to IgM, there is no evidence that they play a role in masking antigenic sites (Stevenson *et al.* 2014). Despite the non-specific binding to IgM (Lambert *et al.* 2014) have shown that naturally acquired opsonizing antibodies to infected erythrocytes expressing var2CSA can be inhibited by incubation with DBL2X, DBL3X or DBL5*ε* domains. The N-terminal NTS-DBL2X region of the molecule is a prime target because antibodies raised to this region effectively block adhesion to CSA (Bigey *et al.* 2011; Bordbar *et al.* 2012; Nunes-Silva *et al.* 2014). Doritchamou *et al.* (2013) have suggested that bivalent vaccine based on this region might be sufficient to provide broad protection. However, the DBL2X region alone can stimulate antibodies in mice that inhibit CSA binding (Bordbar *et al.* 2012) and structural studies have confirmed the location of the CSA-binding region within this domain (Clausen *et al.* 2012).

There appear to be fitness differences in naturally occurring var2CSA. Specific var2CSA sequences appear to be associated with high parasitaemia infections. This may suggest evolution of sequences to evade host antibodies, or that some sequences bind more strongly to chondroitin sulphate A (Rovira-Vallbona *et al.* 2013).

This functional specialization shapes the entire PfEMP1 repertoire. PfEMP1 associated with childhood malaria can be divided into three broad groups, A, B and C defined by upstream (ups) elements A, B and C, respectively (Lavstsen *et al.* 2003). Unlike the majority of *var* genes, those in group C are located in clusters close to centromeres. Diversity of *var* genes is maintained by gene conversion events that occur between non-homologous positions within the genome. As a result the architectures of *var* genes, that is, the combinations and number of various DBL and CIDR domains, are highly variable between genomes (Kraemer and Smith, 2003). Despite this, the group A genes appear to be relatively genetically isolated from the B and C genes. Group A PfEMP1 also tend to be longer and contain more DBL and CIDR domains. In contrast, there is evidence for considerable gene conversion between B and C *var* genes (Kraemer and Smith, 2003; Kraemer *et al.* 2007; Rask *et al.* 2010).

Expression of group A PfEMP1 by the infecting parasite population tends to be associated with infections of children with low immunity. This class of PfEMP1 tends to be recognized first as children are exposed to malaria parasites during the first few years of their lives. Parasites selected *in vitro* for recognition by pooled children's antibodies have a tendency to express Group A PfEMP1 (Jensen *et al.* 2004). Recombinant domains from PfEMP1 are recognized in a hierarchical manner and group A PfEMP1 are the first to be recognized (Cham *et al.* 2009, 2010). Finally, parasite expression of group A-like PfEMP1 is differentially selected against *in vivo* as children develop antibodies against the IE surface (Bull *et al.* 2005*a*; Rottmann *et al.* 2006; Warimwe *et al.* 2009, 2012) (see Table 2). In contrast, the expression of group C *var* genes tends, at least in children from Papua New Guinea, to be differentially elevated in children with asymptomatic infections (Kaestli *et al.* 2006; Falk *et al.* 2009).

**Table 2.** A summary of PfEMP1 expression studies in clinical parasite isolates

Location	References	Patients	Method	Disease severity							Asymptomatic							Age							Antibodies						
				cys2	A	B	C	8	13	H	cys2	A	B	C	8	13	H	cys2	A	B	C	8	13	H	cys2	A	B	C	8	13	H
Brazil	[1]	Adults	DBL*α* tag	+																											
PNG	[2]	Children	qPCR		0	0	0					0	−	+																	
PNG	[3]	Children	DBL*α* tag	0	0	0	0				−	−	0	+				0	0	0	+										
Kenya	[4]	Children	DBL*α* tag	0														0						0	−						+
Kenya	[5–7]	Children	DBL*α* tag	+	+					0	0	0					+	−	−					0	−	−					+
Tanzania	[8]	Children	qPCR		+	+	0					−	−	0					−	0	0										
Tanzania	[9]	Children	qPCR		+	0		+	+																						
Malawi	[10]	Children	qPCR		+	−	0																								
Uganda	[11]	Children	DBL*α* tag	0																											
Mali	[12]	Children	DBL*α* tag	+																											
Gabon	[13]	Children	qPCR		−	+	+																								
Gambia	[14]	Children	qPCR		+	+	0																								
Benin	[15]	Children	MS					+																							
Cameroon	[16]	Children	qPCR, MA		0	0	0	+	+			−	−	0	0	0															

Positive and negative associations are shown as + and − symbol, respectively, lack of association is shown as 0, blank means that the comparison was not done. Four levels of immunity are described: Disease severity refers to comparisons between severe and mild malaria, Asymptomatic refers to comparisons between asymptomatic and symptomatic infection; age refers to whether there was an association with higher age of the parasite donor at the time of infection; antibodies asks whether there was an association between var expression and levels of antibodies to infected erythrocytes. Var expression is described as A, Group A; B, group B; C, group C; 8, DC8; 13, DC13; H, Homogenetiy of the var expression profile i.e. the extent to which a single variant dominates. Methods: DBL*α*tag, an expressed sequence tag approach using cloned and sequenced, PCR amplified tags from the DBL*α* domain; qPCR, quantitative PCR using domain specific primers; MS, Mass spectrometry; MA, microarray.

References for each study are coded as follows: 1. Kirchgatter and Portillo Hdel (2002); 2. Kaestli *et al.* (2006); 3. Falk *et al.* (2009); 4. Bull *et al.* (2005*a*); 5. Warimwe *et al.* (2009); 6. Warimwe *et al.* (2012); 7. Warimwe *et al.* (2013); 8. Rottmann *et al.* (2006); 9. Lavstsen *et al.* (2012); 10. Tembo *et al.* (2014); 11. Normark *et al.* (2007); 12. Kyriacou *et al.* (2006); 13. Kalmbach *et al.* (2010); 14. Merrick *et al.* (2012); 15. Bertin *et al.* (2013); 16. Almelli *et al.* (2014).

This antigenic separation between group A and non-group A PfEMP1 reflects a functional difference between these two broad classes. Recombinant CIDR domains from the ‘head structure’ (i.e. the first DBL and CIDR domain) of non-group A PfEMP1 molecules usually bind to CD36, while those from group A PfEMP1 do not (Robinson *et al.* 2003; Janes *et al.* 2011). Recently, the phenomenon of mitotic recombination that would lead to immense additional PfEMP1 diversity appears to be restricted to non-group A PfEMP1 (Claessens *et al.* 2014). Together these observations are consistent with the idea of a trade-off between function and antigenic novelty shaping the genomic organization of the *var* gene family.

### Exceptions to the molecular ‘rules’ may enable parasites to evade antibodies, prolong infections and cause severe malaria

For the sake of clarity, we have greatly simplified the distinction made above between the three classes of PfEMP1 types A, B and C. There are clear exceptions to these classifications and these exceptions to the norm may be of importance to our understanding of how these molecules evade antibodies. A transitional group between A and B PfEMP1 genes (B/A) exist that have upsB promotors but are otherwise like group A genes (Lavstsen *et al.* 2003) and are predicted not to bind CD36 (Robinson *et al.* 2003). Similarly transitional genes between groups B & C (B/C) are common (Lavstsen *et al.* 2003). The existence of transitional groups indicates that the three classes of genes, cannot be considered as genetically distinct families of molecules.

To refine our understanding of *var* gene organization, Rask *et al.* (2010) developed a system of classification that works from the level of individual homology blocks and domain subclasses to define common structures within *var* genes independent of their upstream promotors. This has greatly expanded our ability to describe similarity and differences between PfEMP1 molecules. The most immediately useful idea has been that of the ‘domain cassette’ (DC). The most prominent DCs are shown in Fig. 2 and Table 1. Two DCs in particular, DC8 and DC13 are of interest because genes containing these were found to be enriched by selecting infected erythrocytes for *in vitro* adhesion to cell lines derived from human brain endothelial cells (Avril *et al.* 2012; Claessens *et al.* 2012) and are expressed in children with severe malaria (Lavstsen *et al.* 2012). Specific CIDR domain subclasses found within these DCs, instead of binding to CD36, are associated with binding to EPCR Adhesion to EPCR has been proposed to cause dysregulation of inflammation in the brain leading to cerebral malaria (Moxon *et al.* 2013; Turner *et al.* 2013). CIDR domains important to EPCR binding contain conserved epitopes close to the EPCR-binding site that are recognized by malaria-exposed children (Lau *et al.* 2015). In terms of vaccine development, these results are encouraging and further support the idea of broad functional specialization within PfEMP1 family.

Overall these studies demonstrate that modularity exists at various levels within the structure of PfEMP1 associated with childhood malaria: at the level of DCs, domain classes and homology blocks. This would potentially enable different functional and antigenic components to exist in varying contexts. Whether this modularity could delay responses to previously encountered functional domains through re-assortment of T-cell and B-cell epitopes is yet to be explored. Buckee and Recker (2012) have suggested that the existence of large numbers of different functional domains on group A PfEMP1 could enable these molecules to support endothelial binding even after antibodies have reversed binding mediated by one or more of the domains. The observation of highly homogeneous expression of group A PfEMP1 in the presence of high levels of antibodies in both severe malaria (Warimwe *et al.* 2013) and asymptomatic infection (Warimwe *et al.* 2012) suggests that these antigenically more conserved variants can survive in individuals with high antibody responses, highlighting the need to fully understand how parasites evade antibodies. We have tended to emphasize the rapid acquisition of immunity to severe non-cerebral malaria (Gupta *et al.* 1999*b*) rather than the fact that cerebral malaria has a slightly later age prevalence than other forms of severe malaria (Greenwood *et al.* 1991; Marsh, 1992; Gupta *et al.* 1999*a*; Roca-Feltrer *et al.* 2010; Griffin *et al.* 2015) and may involve the ability of parasites to effectively escape from the existing antibody responses (see also Box 2).

Box 2. The link between virulence and transmission.

Why are some PfEMP1 variants associated with more virulence than others? One suggestion from evolution of virulence theory, is that a trade-off exists between parasite virulence and effective transmission (Mackinnon and Read, 2004), i.e. that some level of virulence is required for competitive transmission to new hosts. For this reason, Hayward *et al.* (1999) attempted to demonstrate a close mechanistic link by demonstrating what appeared to be normal expression of CD36-binding PfEMP1 on early gametocytes. However, more recent data suggest that the link between the PfEMP1 expression and transmission is indirect since early gametocytes have a very different programme of PfEMP1 expression (Alano, 2014; Ankarklev *et al.* 2014). Recent articles have re-emphasized the link between virulence and transmissibility by demonstrating an increase in both broad *var* gene expression and sexual commitment in parasites carrying deletions for key epigenetic regulator genes PfHP1 (Coleman *et al.* 2014) and PfHDA2 (Brancucci *et al.* 2014*a*). However, it is important to distinguish clearly between mechanistic and evolutionary links between virulence and transmissibility. The PfAP2-G gene recently found to be the gene responsible for immediate control of sexual commitment is flanked by insulator-like pairing elements that are also found in *var* genes, raising the possibility that sexual commitment and *var* gene expression are under common control to ensure their expression is mutually exclusive (Kafsack *et al.* 2014).

### Immunological studies of PfEMP1 domains

Five kinds of question need to be addressed in immunological and epidemiological studies to determine the extent to which different features of *var* genes are targets of naturally acquired immunity to malaria and/or potential vaccine candidates: (1) Are recombinant domains recognized broadly by naturally acquired antibodies or antibodies raised in animals? (2) Do antibodies raised in animals or adsorbed from human serum, bind to the surface of parasite infected erythrocytes, opsonise-infected erythrocytes or reverse cytoadhesion? (3) Is carriage of these antibodies associated with low frequency of future episodes of malaria or low severity of a current infection? (4) Is expression of the PfEMP1 feature by parasites *in vivo* negatively associated with host immunity measured as disease severity, IE antibodies carried at the time of infection or host age? (5) Are there conserved epitopes in PfEMP1 that can be targeted by antibodies? (6) Can antibodies be shown directly to protect against malaria?

Tables 1 and 2 together with Fig. 2 bring together some of the key information that has been gathered in relation to these questions. Considered alone these questions have important limitations. Question 1 does not distinguish between epitopes exposed by living or dead parasites. Question 3 carries the danger of confounding by cumulative exposure. Because of the diversity and immunogenicity of PfEMP1, recognition of various domains is likely to be excellent markers of past exposure, possibly better than the ‘exposure’ controls used.

Parasite expression levels in association with antibodies or age at the time of disease are likely to be a powerful approach to dissect specific components of the naturally acquired immune response. However, expression studies in parasites still rely heavily on indirect methods. Expression of DCs currently relies on qPCR using sets of primers that identify homology blocks within domains commonly encountered within specific DCs. It is important to note that this approach measures expression levels of specific DCs only indirectly and assumes that strong associations between homology blocks is maintained within the parasite population (Lavstsen *et al.* 2012). Further information is needed on the stability of DCs over time and geographical space. To understand the effect of antibody selection on various different sequence features within *var* genes we urgently need complete sequence information from expressed *var* genes from parasites infecting children with different levels of naturally acquired antibodies. This will help us to fully understand why children succumb to life-threatening disease. To this end, a recent study has used mass spectrometry to identify sequence features related to DC8 in children with severe malaria (Bertin *et al.* 2013), but there are still only very few studies that have attempted to place *var* gene expression profiles at the time of severe malaria within the context of the immune response (Table 2).

### A summary of attempts to directly address the challenge of developing a PfEMP1-based vaccine to childhood malaria

In this section, we will summarize the progress that has been made in direct attempts to address question 5 above and protect animals through vaccination with PfEMP1.

#### CD36-antibodies to CIDR

First attempts to raise cross-reactive antibodies to PfEMP1 focused on CD36-binding CIDR domains. The rationale for this was that the CIDR region, known to bind what was at the time the most prominent parasite receptor (CD36) was shown to be immunogenic and the target of antibodies that agglutinate parasites (Baruch *et al.* 1997). In the first study done through vaccination of *Aotus* monkeys with recombinant CD36-binding CIDR, there was cross-agglutination, and reactivity to recombinant CIDR, but this was not supported by surface labelling or reversal of CD36 binding (Gamain *et al.* 2001). Using the *Aotus* Monkey model, Baruch and colleagues subsequently demonstrated that immunization with a single short region of the CD36-binding region of a single CIDR [r179 from the ‘Malayan Camp’ laboratory parasite line (Baruch *et al.* 1997)] can immunize against infection with the homologous strain (Baruch *et al.* 2002). This seemed to suggest that ‘determinant spreading’ (Lehmann *et al.* 1993) was occurring and that immunization with a non-immunodominant region boosts the development of cross-reactive antibodies. However, in this model in which monkeys were challenged directly with ring stage parasites, there is a clear first wave of infection that is dominated by a single variant, which may not model sporozoite challenge of humans (Wang *et al.* 2009). This may help explain why the vaccination did not protect against heterologous parasite isolates (Baruch *et al.* 2002). To favour the stimulation of cross-reactive antibodies, simultaneous vaccination of mice with three different proteins [MC CIDR1 (residues 1–267), FVO CIDR1 (residues 1–260) and A4tres CIDR1 (residues 1–262)] was used. This led to an increase in cross-recognition over separate vaccination (Gratepanche *et al.* 2003). However, such a regime did not protect *Aotus* from the virulent line FVO, despite some protection from anaemia (Makobongo *et al.* 2006).

#### Antibodies to DBLalpha

DBLalpha domains are present within all PfEMP1 variants associated with childhood malaria and are therefore an attractive domain to target in a vaccine. However, raising antibodies that recognize the surface of infected erythrocyte has been a challenge. Initial studies on immunization with *Escherichia coli* expressed DBLalpha region lead to antibodies that recognize conserved regions of recombinant proteins but that do not recognize the surface of the infected erythrocytes (Oguariri *et al.* 2003; Chen *et al.* 2004). This problem was overcome by immunization with mini-*var* Semliki forest virus constructs that are displayed on the surface of virus-infected cells. The effectiveness of antibodies in reversing rosetting was tested using antibodies against DBLalpha from FCR3S1·2var1 (Chen *et al.* 2004) and a rat lung *in vivo* sequestration model was used to test for reversal of sequestration. The *in vivo* model using ^99m^technetium-labelled iRBC was also adapted for use in cynomolgus macaques and shown to be effective (Moll *et al.* 2007) and led to a 46% reduction in sequestration against the homologous isolate. However, the *var* gene used in these studies was subsequently shown not to be involved in rosetting (Albrecht *et al.* 2011) and the authors suggest that anti-rosetting activity was due to cross-reactivity.

The development of platforms for controlled human infection with malaria parasites are likely to allow rapid testing of vaccine candidates that has not been possible previously (Hodgson *et al.* 2014; Obiero *et al.* 2015).

### Global approaches to finding important protective epitopes on PfEMP1

Using an entirely different approach, Blomqvist *et al.* (2013) used *var* gene expression data of parasites from Ugandan children to identify short stretches of PfEMP1 sequence that are associated with severe malaria (Normark *et al.* 2007). They raised antibodies to peptides based around these motifs. One of these peptides called RDSM (respiratory distress severe malaria) containing the ALNRKE motif and associated with respiratory distress, stimulated production of antibodies that cross-reacted against several parasite lines including R29, whose expressed *var* gene (IT4 var9) contains the very similar AINRKE motif (Blomqvist *et al.* 2013). This region is in a relatively conserved part of the molecule in the same region as another peptide from var2CSA that is associated with changes in sequence expression between primigravid and multigravid women (Dahlback *et al.* 2006).

#### Directing response to the conserved regions

As is the case with other highly diverse pathogens naturally acquired immune responses are most frequently directed towards the diverse immunodominant regions despite rare individuals who make substantial responses to conserved regions (Rathore *et al.* 2014). In HIV, researchers are trying to tackle this problem by directing the immune response to the most conserved regions of the surface antigens. This they do by bringing together artificially the conserved regions of antigens into one composite antigen (Hanke, 2014). A similar approach has been explored for PfEMP1 by Patarroyo *et al.* (2014) who have initiated a high-throughput approach to screening 15–20aa long peptides for host cell binding [high activity binding peptides (HABPs)], immunogenicity and protective efficacy in *Aotus*. Their approach involves modifications of otherwise non-immunogenic regions within a single *var* gene (var2CSA from Dd2) to convert them into immunogenic peptides [modified HABPs (mHABPs)]. The most promising peptides are both located within homology block 4 (Rask *et al.* 2010) and contain the GACxPxRRxxLC canonical motif (Patarroyo *et al.* 2014).

### Future directions

There have been some exciting recent developments in our understanding of the PfEMP1 family of surface antigens, but there are clearly challenges ahead.

Despite the plausible mechanism that would explain the pathology of cerebral malaria (Taylor *et al*. 2013) it is still unclear whether EPCR is a common receptor for parasites since only a relatively small number of clinical parasite isolates have so far been tested. A recent paper by Esser *et al.* (2014) suggests the existence of a large number of possible novel host receptors for PfEMP1; however, it is not clear if any of these could sustain an infection at high parasitaemia.

Two recent reports suggest that on the one hand *var* expression may be under some level of global control (Merrick *et al.* 2012) and on the other hand, that two other gene families may be able to support sequestration (Niang *et al.* 2014; Goel *et al.* 2015). PfEMP1 appears also to be partially controlled at the translational level (Brancucci *et al.* 2014*b*). This raises the possibility that the parasite could diversify both the type and total amount of variant antigen expressed on the infected erythrocyte surface.

The important question here is whether the level of sequestration supported by STEVOR and RIFINs in the absence of PfEMP1 could support parasite loads associated with severe malaria. Though knockdowns of *var* have an important effect on the antigenic properties of laboratory isolates (Chan *et al.* 2012), they may not fully model the behaviour of non-PfEMP1 VSA *in vivo*. Warimwe *et al.* (2012) observed an association between respiratory distress and rosetting in Kenya. Despite a strong overall association between rosetting and the expression of group A *var* genes, the observed association was independent of group A-like *var* gene expression or any other subgroup of *var* genes that could be identified. One possible interpretation of this is that the association between rosetting and respiratory distress was driven by a subset of parasites for which rosetting is mediated through RIFINs or STEVOR. This highlights the need for full sequence information of VSA expressed under different levels of naturally acquired immunity.

Future research on developing interventions based on PfEMP1 needs to continue to refine definitions of different cytoadhesive phenotypes at the molecular level. Various terms such as ‘rosetting’, ‘VSA_SM_’, ‘group A PfEMP1’, ‘DC8’, in the same way as clinical definitions such as ‘cerebral malaria’ (Taylor *et al.* 2004), are essential for generating hypotheses, but what they describe are potentially heterogeneous. More studies are still needed to directly link structure, cytoadhesive function and antigenicity with naturally acquired immunity to clearly defined malarial disease.
